# Estimating Heritabilities and Breeding Values From Censored Phenotypes Using a Data Augmentation Approach

**DOI:** 10.3389/fgene.2022.867152

**Published:** 2022-07-25

**Authors:** Melissa A. Stephen, Hao Cheng, Jennie E. Pryce, Chris R. Burke, Nicole M. Steele, Claire V. C. Phyn, Dorian J. Garrick

**Affiliations:** ^1^ DairyNZ Ltd., Hamilton, New Zealand; ^2^ AL Rae Centre for Genetics and Breeding—Massey University, Hamilton, New Zealand; ^3^ Department of Animal Science, University of California, Davis, Davis, CA, United States; ^4^ Centre for AgriBioscience, Agriculture Victoria Research, AgriBio, Bundoora, VIC, Australia; ^5^ School of Applied Systems Biology, La Trobe University, Bundoora, VIC, Australia

**Keywords:** MCMC, gibbs sampling, baysian, data augmentation, censored, breeding

## Abstract

Time-dependent traits are often subject to censorship, where instead of precise phenotypes, only a lower and/or upper bound can be established for some of the individuals. Censorship reduces the precision of phenotypes but can represent compromise between measurement cost and animal ethics considerations. This compromise is particularly relevant for genetic evaluation because phenotyping initiatives often involve thousands of individuals. This research aimed to: 1) demonstrate a data augmentation approach for analysing censored phenotypes, and 2) quantify the implications of phenotype censorship on estimation of heritabilities and predictions of breeding values. First, we simulated uncensored phenotypes, representing fine-scale “age at puberty” for each individual in a population of some 5,000 animals across 50 herds. Analysis of these uncensored phenotypes provided a gold-standard control. We then produced seven “test” phenotypes by superimposing varying degrees of left, interval, and/or right censorship, as if herds were measured on only one, two or three occasions, with a binary measure categorized for animals at each visit (either pre or post pubertal). We demonstrated that our estimates of heritabilities and predictions of breeding values obtained using a data augmentation approach were remarkably robust to phenotype censorship. Our results have important practical implications for measuring time-dependent traits for genetic evaluation. More specifically, we suggest that data collection can be designed with relatively infrequent repeated measures, thereby reducing costs and increasing feasibility across large numbers of animals.

## Introduction

Maximizing the number of individuals contributing phenotypes to an analysis is particularly important in genetic evaluation and selection. Accuracy of evaluation and selection intensity are two key drivers of genetic improvement in a population (Rendel and Robertson, 1950). For any given trait, the accuracy of an individual’s estimated breeding value (EBV) will improve as more of its immediate descendants have phenotypes measured. Response to selection depends on the EBV superiority of the individuals that are selected to become parents (Rendel and Robertson, 1950). In selection schemes that include individual phenotypes on selection candidates, selection intensity increases as more animals have phenotypes measured. However, precise measurement of phenotypes across large numbers of individuals can be problematic, especially when they are expensive to measure, require invasive procedures and/or measures must be repeated over time. Censored phenotypes are easier and cheaper to obtain, as fewer, and/or less specific observations are required. It follows that where resources are limited, the strategic use of censorship can enable researchers to phenotype considerably more individuals.

There are several situations where animal breeders deliberately censor phenotypes. First, when a continuous trait, such as shoulder height, is measured using ordinal categories (for example a score of 1–9) instead of the underlying continuous variable. This type of censoring of continuous phenotypes makes them easier and faster to measure. Kizilkaya et al. (2014) reported that although EBV accuracy was compromised by this approach, it could be overcome by roughly doubling the number of animals phenotyped. A second situation is when a time-dependent trait is measured at relatively infrequent intervals. For example, Fortes et al. (2012) measured puberty status at intervals of four to 6 weeks, in preference to sustaining the cost and ethical issues of daily measures. In addition to interval censoring, left and right censoring are often introduced as a means to reduce observations. Left censoring occurs when the observation window begins after some animals have already expressed their phenotype, whereas right censoring occurs when animals express their phenotype after the observation window closes. Longevity is a phenotype that is subject to right-censoring, because individuals that are still alive at the time of data collection will only have a lower bound observation (Ducrocq et al., 1988).

Where time-dependent traits, such as age of puberty, are subject to left and interval censoring, individuals are often assigned a phenotype based on their age when they were first observed to have reached the threshold criterion (for example, Fortes et al., 2012). That logic cannot be applied to right censoring, as there is essentially no upper bound on an animal’s phenotype. A number of methods have been developed for handling right-censored phenotypes. Two common examples include adding an arbitrary penalty for right-censored phenotypes, or predicting them using survival analysis techniques. Donoghue et al. (2004) analysed conception phenotypes by adding a 21 days penalty to right-censored phenotypes, while Ducrocq et al. (1988) analysed longevity phenotypes using survival analysis techniques such as the Cox proportional hazard model and the Weibull model to predict right-censored phenotypes. A data augmentation method (Tanner and Wong, 1987), where the phenotypes of any censored individuals are sampled from a truncated predictive distribution, provides an alternative approach for handling censored data. Donoghue et al., (2004) compared penalty and data augmentation approaches in their analysis of right-censored conception phenotypes and found that the results were similar.

It is likely that left, right and interval censorship of time-dependent traits may compromise the accuracy of EBVs. That said, high EBV concordance reported across varying degrees of right-censoring (Guo et al., 2001; Donoghue et al., 2004) indicates that this compromise may be minimal. It is difficult to investigate the implications of phenotype censorship for traits that are commonly left, interval and right-censored, such as age of puberty, because the cost of obtaining precise phenotypes for an “uncensored” comparison is prohibitive. Instead, we have simulated precise phenotypes, representing the trait “age at puberty” (AGEP) and then applied a range of censorship scenarios to these phenotypes. These results can be used to make inferences about time-dependent traits that are challenging to measure precisely.

The aims of this study were to: 1) demonstrate a data augmentation approach for analysing left, interval and right-censored data, and 2) quantify the implications of varying phenotype censorship on estimates of heritabilities and predictions of EBVs, using a categorical, time-dependent trait. Our hypothesis was that the heritability and EBV rankings would be robust to phenotype censorship.

## Methods

### Simulated Phenotypes

We used the software XSim (Cheng et al., 2015), implemented in Julia (Bezanson et al., 2017) to simulate precise phenotypes representing the trait AGEP. The phenotypes were simulated using real single-nucleotide polymorphism (SNP) genotype data (Weatherbys Versa 50 k SNP array) from 4,935 Holstein-Friesian, Holstein-Friesian cross Jersey cows. These 4,935 cows were born in 2018 and represent around 260 sires. We carried out quality control on these SNP genotypes prior to our simulation, disregarding unmapped SNP, as well as 2,120 SNP with minor allele frequency <1%. This left around 47,000 SNP included in our analyses. We simulated a phenotype that represented AGEP, by specifying a genetic variance of 297 days, and heritability of 0.33 (Dennis et al., 2018). Animals were randomly assigned to one of 50 contemporary groups. The mean of each contemporary group was sampled at random from a normal distribution, with a mean of 342 days (Dennis et al., 2018) and a variance of 20. We assumed AGEP to be polygenic, with 500 SNP loci spread across the genome chosen to represent simulated additive QTL. The resultant precise phenotypes provided data for our “gold-standard” (GOLD) control analyses.

**FIGURE 1:**
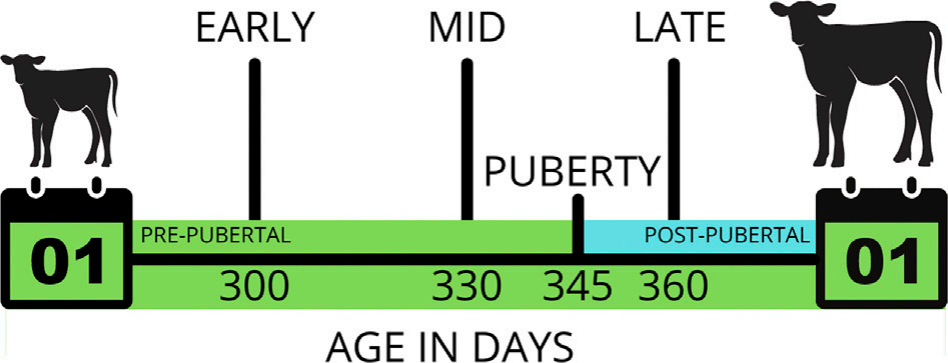
An example of interval censoring for “age at puberty”. If this animal was observed daily, it would be recorded as attaining puberty at 345 days old. However, if the herd was observed only three times (early, mid and late), when this animal was 300, 330 and 360 days old, respectively, its phenotype would fall within the bounds of 330–360 days.

We superimposed varying degrees of censorship to simulate these animals being observed at only 1, 2 or 3 herd visits for a seasonal window during which they would have been expected to attain puberty. In the first censored scenario, three herd visits (early, mid and late; EML) were simulated for each herd. The mid observation was on the day where 50% of the herd had attained puberty, and the early and late observations were 20 days either side of that day. This timing resulted in an even number of animals with left, interval and right censoring. In the second to fourth censored scenarios, herd visits were restricted to just the early and mid (EM), the mid and late (ML), or the early and late (EL) visits. In the fifth to seventh censored scenarios, there was only one visit to each herd, with an early only (E), a mid only (M) or a late only (L) visit. Under censorship, the continuous variable GOLD was unobserved. Instead, the phenotype for each animal was only known to fall within a lower and/or upper bound (Figure 1).

### Data Augmentation

**FIGURE 2:**
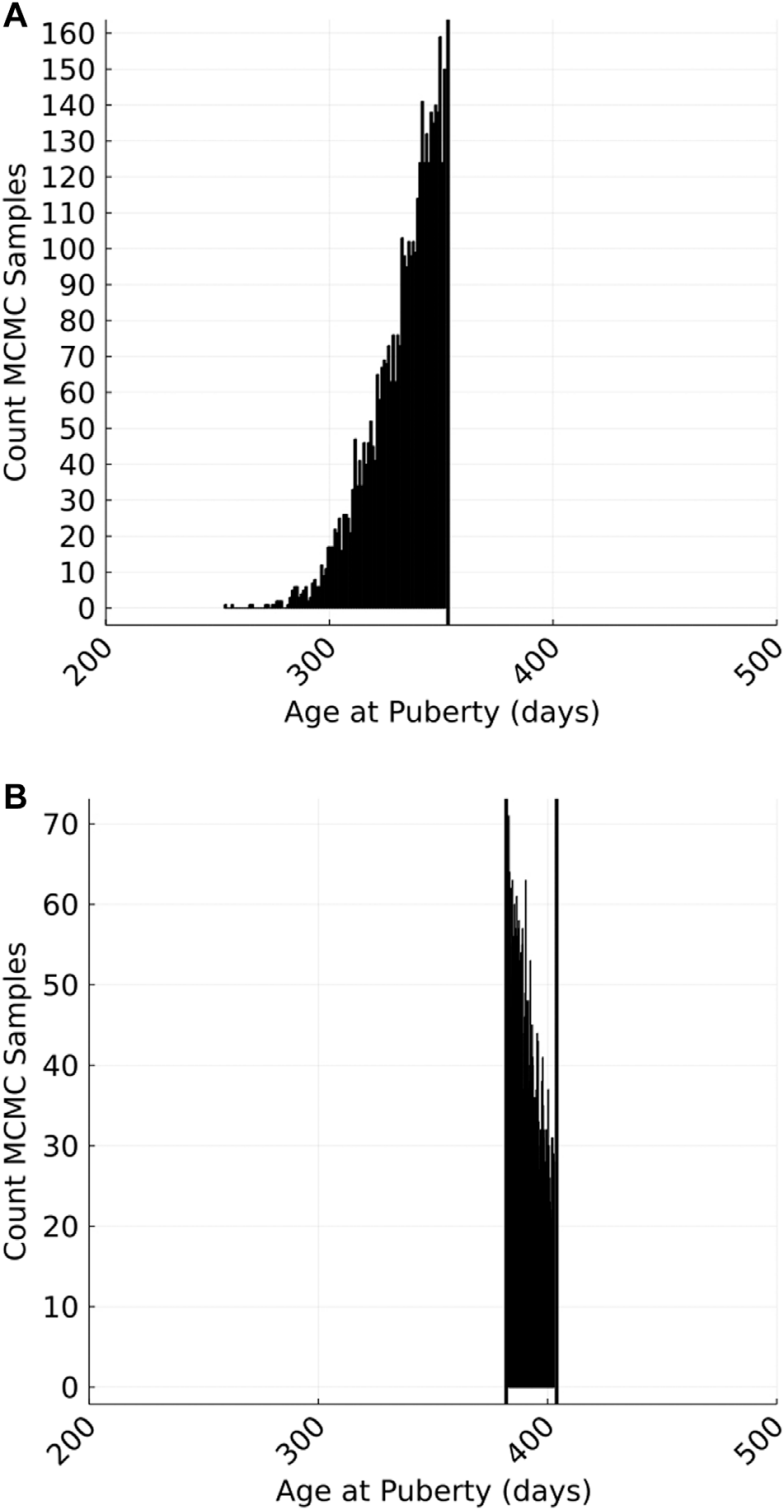
An example of the Markov-chain Monte Carlo (MCMC) sampled phenotypes for a single animal with a left- **(A)** or interval- **(B)** censored phenotype. Left censoring occurs where an animal has attained puberty on or before the first herd visit (i.e., sampled phenotypes are truncated by the age of the animal on the first visit). Interval censoring occurs when an animal reaches puberty between two visits (i.e., sampled phenotypes are truncated by the age of the animal on the flanking visits.

We used a Markov-chain Monte Carlo (MCMC) technique that included data augmentation (Tanner and Wong, 1987) to obtain posterior distributions for variance parameters and EBVs from censored phenotypes. The unobserved continuous variables representing the actual age that each animal attained puberty was treated as an unknown variable (hereinafter referred to as liabilities) whose value must fall between a known upper and lower bound. Plausible AGEP phenotypes were repeatedly sampled from a truncated predictive distribution for each animal. The sampled phenotypes were continuous variables representing a plausible value for each animal’s AGEP, even though the observations on which they were based were binary (pre- or post-pubertal on a given herd visit). The mean and variance of these predictive distributions were determined by the simultaneous sampling of fixed herd effects and marker effects (mean) and residual variance (variance) within a single site Gibbs sampling approach. The truncation points for each predictive distribution were the known upper and/or lower bounds for each animal. This MCMC approach produced a posterior distribution of AGEP phenotypes for each animal (Figure 2).

### Model Equation

We fitted a mixed linear model using Bayesian methodology *via* single-site Gibbs sampling to construct a Markov chain of plausible values of unknowns. The model included random marker effects using BayesC priors (Garrick et al., 2014). Briefly, marker effects were assumed to follow a mixture distribution where their effects were either zero, with prior probability Pi = 0.99, or independently normally distributed with mean 0 and constant variance, with prior probability (1-pi). Given the mixture prior, at each iteration of the Gibbs sampler effects for about 470 markers were sampled from the non-zero distribution to collectively explain the breeding value or genetic component of the phenotypic differences between the 5,000 animals in our simulated dataset. We sampled the AGEP phenotypes conditional on the current sample values for all the effects in the linear model, and then fitted the linear model conditional on the sample of AGEP phenotypes, and so on.

The resultant MCMC samples of effects represented fixed herd effects, marker effects, and variance parameters. Matrix representation of the linear marker effects model equation is:

y=Xb+Ma+e (1)

where **y** is a vector of phenotypes (one phenotype per study animal), **b** is a vector of herd effects, **a** is a vector of additive marker effects. The vector **e** represents residuals corresponding to each of the phenotypes. The residuals are assumed to be independently normally distributed, with homogeneous residual variance. The incidence matrix **X** relates each phenotype record to relevant fixed herd effects. The covariate matrix **M** relates each phenotype record to the number of one of the alleles at each SNP marker. The matrix **M** has a column for each SNP marker, and a row for each phenotype.

In all analyses, the unknowns include the vectors **b** and **a** and the scalars representing genetic and residual variances. Where phenotypes are censored with known lower or upper bounds, the vector **y** is also unknown, except for the bounds on each observation.

### Software and Solver

We used command line bash scripts and Julia (Bezanson et al., 2017) packages CSV, StatsPlots, and DataFrames to pre-process observation data into the vectors representing control or censored phenotypes. We performed the genetic analyses using the JWAS package (Cheng et al., 2018). The MCMC comprised 50,000 samples, with the first 10,000 samples disregarded as a burn-in and then every 10th sample of the MCMC was retained. The Julia environment was used to post-process the results. We produced credibility intervals for genetic variance, residual variance, and heritability for the 5% (lower bound) and 95% (upper bound) percentiles based on all post-burn in samples. We used two methods to test for evidence of non-convergence of our MCMC chains. First, we undertook the diagnostic test described by Geweke (1992), and second, we observed trace plots to visually assess the convergence of posterior means of each parameter.

### Comparisons Across Censorship Scenario

We used Pearson’s correlation coefficient to quantify the extent of re-ranking between EBVs obtained from each of our censorship scenarios. Correlations of EBVs included all animals with simulated phenotypes (*n* = 4,935).

## Results

Correlations between the posterior mean of MCMC phenotypes sampled for each animal and the control (GOLD) phenotype were strong and positive across all censorship scenarios (Table 1). Strong correlations were observed between EBVs estimated using phenotype bounds from each censorship scenario and EBVs estimated using GOLD phenotypes (Table 1). Unsurprisingly, these correlations decreased as censorship increased. Where the censorship scenario included at least two herd visits (EML, EM, ML, or EL), correlations between the GOLD and estimated phenotypes ranged from 0.90 to 0.95 and those between EBVs ranged from 0.92 to 0.96. Where the censorship scenario included just one herd visit (E, M or L), correlations between the GOLD and estimated (i.e., censored) phenotypes ranged from 0.81 to 0.85 and those between EBVs ranged from 0.85 to 0.88.

**TABLE 1:** Comparison across censorship scenarios for simulated “age at puberty” phenotypes. Correlations between phenotypes (*n* = 4,935) (white shading, below diagonal), correlations between EBVs (*n* = 4,935) (grey shading, above the diagonal), and heritabilities with 90% credibility intervals (bold, on the diagonal). In the control scenario (GOLD), the phenotypes represented those that would be obtained when animals were observed daily. Censored scenarios simulate if animals in a herd were observed at either one, two or three visits. In the first censored scenario, three herd visits (early, mid and late; EML) were simulated for each herd. In the second to fourth censored scenarios, herd visits were restricted to just the early and mid (EM), mid and late (ML), or early and late (EL) visits. In the fifth to seventh censored scenarios, herd visits were restricted to one per herd, with an early only (E), a mid only (M) or a late only (L) visit. 90% credibility intervals did not exceed 0.02 for any of the correlations.

	GOLD	EML	EM	ML	EL	E	M	L
GOLD	**0.29 (0.26,0.31)**	0.96	0.92	0.93	0.94	0.85	0.88	0.85
EML	0.95	**0.30 (0.27,0.33)**	0.96	0.96	0.98	0.89	0.91	0.88
EM	0.90	0.95	**0.29 (0.26,0.33)**	0.93	0.91	0.92	0.95	0.78
ML	0.91	0.95	0.90	**0.29 (0.26,0.33)**	0.91	0.80	0.95	0.91
EL	0.93	0.98	0.90	0.90	**0.30 (0.27,0.33)**	0.90	0.83	0.89
E	0.81	0.86	0.9	0.71	0.88	**0.27 (0.22,0.32)**	0.80	0.72
M	0.85	0.90	0.95	0.94	0.80	0.73	**0.29 (0.25,0.34)**	0.79
L	0.81	0.86	0.70	0.90	0.88	0.58	0.72	**0.27 (0.22,0.32)**

Correlations between the posterior means of sampled phenotypes from the EML censorship scenario and other censored scenarios ranged from 0.86 to 0.98 (Table 1). Likewise, correlations between EBVs from the EML censorship scenario and EBVs from the other more censored scenarios were all strong and positive, ranging from 0.88 to 0.96 (Table 1).

The posterior mean for the heritability of AGEP estimated using GOLD phenotypes was 0.29 (Table 1). The posterior mean of estimated heritabilities for different censorship scenarios also tended to be around 0.29 with 90% credibility intervals ranging from 0.22 to 0.34.

## Discussion

We determined that a data augmentation approach to analysing left-, interval- and right-censored data resulted in precisely estimated phenotypes for a time-dependent categorical trait, using simulated AGEP phenotypes as a case study. The extent of animal re-ranking, indicated by comparing correlations between censored phenotypes and their precise phenotypes (control), was relatively low even under extreme censorship scenarios, where animals only had a single observation. Furthermore, EBVs were robust to phenotype censorship and animal rankings were largely consistent with our gold standard control scenario. In particular, the correlations between control EBVs and EBVs from any censored scenario where there were at least two visits per herd were greater than 0.90. We also determined that heritability estimates were relatively unaffected by phenotype censoring; across both control and censored scenarios, heritabilities tended to be around 0.29. Previous studies focusing on the implications of right censoring also indicated concordance of EBVs across varying degrees of censorship (Guo et al., 2001; Donoghue et al., 2004) and that a data augmentation approach produced minimal differences in variance parameters compared with uncensored phenotypes (Sorensen et al., 1998; Donoghue et al., 2004). Together, these outcomes support our hypothesis that heritabilities and EBVs can be robust to phenotype censorship. In the analysis presented here we have fit SNPs directly using a marker effects model. That said, our data augmentation approach can be extended to a wide range of models including those analyses where a genomic or pedigree relationship matrix is used to describe the variance-covariance matrix between individuals.

Intentional phenotype censorship is useful for reducing cost and/or animal welfare concerns associated with phenotype collection. This is especially true for time-dependent traits, where repeated measurements are required to produce precise phenotypes. The trait AGEP provides a good case study as all animals begin pre-pubertal and, over time, reach sexual maturity and become post-pubertal. The timing of puberty varies between individuals and is influenced by a range of genetic and environmental factors. Puberty status can be determined through behavior monitoring, ovarian ultrasonography and/or blood testing for plasma progesterone concentrations (Morris et al., 2000; Macdonald et al., 2007; Handcock et al., 2021); however, these measurements are labor intensive and therefore costly, in addition to being somewhat invasive, potentially compromising animal welfare or raising ethical issues. Hence, although daily observations would yield a precise phenotype, AGEP is often measured using as few observations as possible, resulting in censored phenotypes (Hickson et al., 2011; Fortes et al., 2013; Handcock et al., 2021). Here, our censored scenarios simulated relatively infrequent herd visits around the time that animals would be expected to attain puberty. Our results provide support for the strategic use of phenotype censoring, indicating that the effects on heritabilities and EBVs may be inconsequential for a time-dependent trait like AGEP and other commonly censored traits, such as longevity and/or other fertility phenotypes.

The current analysis has not exhaustively considered the implications of timing of observations. We timed our simulated herd visits to obtain about 25% of animals with left, interval E to M, interval M to L and right censoring using knowledge of the median AGEP for each herd. In reality, that information is not available in advance to plan the timing of observations. As fewer herd visits are undertaken, timing may become more important. For example, if there is only one visit per herd, and it occurs earlier or later than the day of median AGEP there will be less variation among observations. In the worst case, all animals may be yet to reach puberty, or all animals may be post pubertal. Their phenotypes would not add value to genetic analysis, as any animal effects would be entirely confounded by the herd effect. We have investigated the implications of visit timing within the bounds of our visit schedule. For example, our E scenario represents a single early visit, while our L scenario represents a single late visit. Our results indicate strong correlations between EBVs produced by all three scenarios with two herd visits. Therefore, when there are at least two visits, the timing is less important. Conversely, the correlations between EBVs produced by our three single visit scenarios are slightly weaker, indicating that when there is only one herd visit, animal selection decisions may be materially altered depending on visit timing. Further investigation is required to quantify the accuracy of EBVs produced using phenotypes from different visit timing before a recommendation on optimal timing can be made for a specific trait. If a visit was earlier than desirable, and most if not all animals were pre pubertal, there is still the option of visiting again to capture more information. A similar option is not available if a visit was later than desirable.

Based on the case study presented here, we conclude that heritability and EBV estimations for categorical, time-dependent traits are likely to be robust to left- interval- and right-censorship of phenotypes. In regard to the design of phenotyping strategies for specific traits, further simulation may be warranted. We assumed that once an animal attained puberty, she would be observed as pubertal thereafter with little measurement error. However, in reality, a trait like age at puberty could incur a relatively high incidence of false negative measures (where the animal has reached or exceeded the threshold but is observed to be under threshold). The extent of errors in allocating a phenotype to an animal would have implications on optimal measurement design. Further, we investigated only a single trait heritability, but the simulation could be applied to phenotypes with varied heritabilities, such that the implications of censorship on very low or very high heritability traits might also be quantified.

## Data Availability

The original contributions presented in the study are included in the article/Supplementary Material, further inquiries can be directed to the corresponding author.
